# Can admission lipoprotein-associated phospholipase A2 predict the symptomatic cerebral vasospasm following aneurysmal subarachnoid hemorrhage?

**DOI:** 10.1186/s41016-020-00188-z

**Published:** 2020-04-04

**Authors:** Chen-Yu Ding, Fang-Yu Wang, Han-Pei Cai, Xiao-Yong Chen, Shu-Fa Zheng, Liang-Hong Yu, Yuan-Xiang Lin, Zhang-Ya Lin, De-Zhi Kang

**Affiliations:** Department of Neurosurgery, The First Affiliated Hospital of Fujian Medical University, Fuzhou, Fujian People’s Republic of China

**Keywords:** Aneurysmal subarachnoid hemorrhage, Vasospasm, Biological markers

## Abstract

**Background:**

Inflammation has been believed to be related to the development of cerebral vasospasm following aneurysmal subarachnoid hemorrhage (aSAH). A potential biomarker for vascular inflammation that is well recognized is the lipoprotein-associated phospholipase A2 (Lp-PLA2). However, whether Lp-PLA2 can predict the occurrence of symptomatic cerebral vasospasm (SCV) in aSAH patients is still unknown. Thus, this study aimed to assess the value of Lp-PLA2 for predicting SCV in patients with aSAH.

**Methods:**

Between March 2017 and April 2018, we evaluated 128 consecutive aSAH patients who were admitted in the First Affiliated Hospital of Fujian Medical University. Their Lp-PLA2 level was obtained within 24 h of the initial bleeding. Factors might be related to SCV were analyzed.

**Results:**

Compared to patients without SCV, those with SCV (9.4%, 12/128) had significantly higher Lp-PLA2 level. Multivariate logistic analysis revealed that worse modified Fisher grade (OR = 10.08, 95% CI = 2.04–49.86, *P* = 0.005) and higher Lp-PLA2 level (OR = 6.66, 95% CI = 1.33–3.30, *P* = 0.021) were significantly associated with SCV, even after adjustment for confounders. Based on the best threshold, Lp-PLA2 had a sensitivity of 83.3% and a specificity of 51.7% for predicting SCV, as shown by the receiver operating characteristic curve analysis. In the poor World Federation of Neurosurgical Societies grade patient sub-group, patients with Lp-PLA2 > 200 μg/L had significantly higher SCV rate than that of patients having Lp-PLA2 ≤ 200 μg/L.

**Conclusion:**

The admission Lp-PLA2 level might be a helpful predictor for SCV in aSAH.

## Background

One of the most frequent and worst complications of aneurysmal subarachnoid hemorrhage (aSAH) is cerebral vasospasm (CV), which leads to the highest mortality in aSAH patients [1]. CV of aSAH could lead to cerebral ischemia or infraction, which worsens patient condition. Further development of ischemia is possible if the condition is not controlled in time, and it could result in delayed ischemic neurological deficits that threat the life of patient [2, 3]. Therefore, it is of clinical significance to understand factors associated with development of CV, as they may help clinicians to identify individuals at risk and potentially prevent this complication.

Currently, clinical rating scales such as the World Federation of Neurosurgical Societies (WFNS) grade and modified Fisher grade, which are easy to use and have relatively high predictive power, are used for assessing the performance of neurological functions and predicting the occurrence of CV [4–6]. However, these clinical scales are not able to differentiate the physical condition of a patient when the changes are small. For example, it is possible for the Fisher scale to categorize all thick clots as grade III, therefore fails to differentiate increasing thickness of subarachnoid clots [7]. To overcome this disadvantage, a number of alternative grading schemes have been proposed. However, each scheme is associated with certain limitations [6–11]. Currently, there is still an unmet need for reliable biomarkers that could be used for more effective diagnosis and assessment of aSAH [12–14] in the clinical setting.

The onset of CV has a complicated mechanism involving many factors. Studies have shown that inflammatory response and inflammatory factors are critically involved in every step of the CV development cascade. Lipoprotein-associated phospholipase A2 (Lp-PLA2) is an available potential biomarker of inflammation in blood vessels [15–18]. It has been reported that in aSAH patients, elevated Lp-PLA2 level is related to angiographic cerebral vasospasm (ACV) [19]. However, whether Lp-PLA2 can predict the occurrence of symptomatic cerebral vasospasm (SCV) in aSAH patients is still unknown. Results from previous studies regarding predictors of CV after aSAH are inconsistent [20], which is mainly caused by using different definitions or diagnostic criteria for CV. For example, both ACV and SCV have been used to define CV [1, 21], but the associated morbidity and mortality of SCV could be higher than those of ACV in aSAH patients. Therefore, this study aimed to investigate the predictive value of Lp-PLA2 level for SCV following aSAH.

## Methods

### Study population

Based on a previous single-center prospective and observational cohort study [22], this study is also observational and prospective, but with additional patients enrolled. Enrollment included aSAH patients admitted to our department between March 2017 and April 2018, but only those consecutive patients who had their post-aSAH Lp-PLA2 level measured within the first 24 h were included for analysis.

The following inclusion criteria were used [22]: (1) admitted to the hospital within 24 h of SAH onset; (2) SAH was caused by intracranial aneurysm as confirmed via computed tomography angiography (CTA) or digital subtraction angiography (DSA); (3) aneurysm was treated, either with clipping or interventional method, within 48 h of admission; and (4) blood Lp-PLA2 level at admission was obtained. The following exclusion criteria were used: (1) patient had surgery or severe infection within the past month; (2) previous occurrence of SAH or had other neurological diseases such as ischemic stroke, hemorrhagic stroke, or severe head trauma; (3) previous use of antiplatelet and anticoagulant drugs or had a history of immunotherapy; and (4) other systemic diseases such as autoimmune disease, uremia, cirrhosis, cancer, chronic lung diseases, and chronic heart diseases including coronary artery diseases and myocardial infarction.

The study protocol was designed in accordance with guidelines outlined in the Declaration of Helsinki and approved by the Ethics Committee of the First Affiliated Hospital of Fujian Medical University (Fujian, China). Informed consent was obtained from the patients or their authorized legal representative if patients cannot sign the form themselves.

### Patient management

The guidelines of American Heart Association and American Stroke Association were used for clinical management [23]. The guideline of Neurocritical Care Society was used for critical care management [24]. Since the day of hospitalization, all patients received nimodipine, either orally (6 × 60 mg/day) or intravenously (2 mg/h).

### Clinical and radiological variables

Medical history, admission conditions, imaging data, prior treatment, and relevant clinical information were collected for every patient. The amount of subarachnoid blood presented on the admission CT image was semi-quantified with the modified Fisher grade. The severity of aSAH was evaluated using the initial Hunt & Hess scale, Glasgow coma scale (GCS), and WFNS grade. Each patient was assigned a grade, which was the average score given by two doctors. If a large deviation was seen, a third, more senior doctor will determine the final score. Good grade was defined as admission WFNS grade < 3, and poor grade was defined as admission WFNS grade ≥ 3.

Lp-PLA2 level at admission was measured during the routine patient care procedure. After patient admission, venous blood was drawn and stored in anti-coagulation tube with ethylenediaminetetraacetic acid. The collected blood was centrifuged at room temperature at 1000*g* for 20 min. The supernatant was collected and stored in – 80 °C freezer until analysis. To measure the plasma Lp-PLA2 level, double-antibody sandwich enzyme-linked immunosorbent assay (ELISA) was performed in this study (Cloud-Clone Corp, Houston, TX, USA), following the protocol provided by the manufacturer. Detection limit of the kit was 0.286 μg/L, coefficient of variation (COV) within the assay was < 10%, and intra-assay COV was < 12%. Lp-PLA2 level > 200μg/L indicated moderate vascular inflammation [25]. Good Lp-PLA2 level was defined as admission Lp-PLA2 level < 200 μg/L, and poor level was defined as admission Lp-PLA2 level ≥ 200 μg/L.

### Detection of cerebral vasospasm

Detection of cerebral vasospasm using computed tomography angiography (CTA) (Acquilion ONE, Toshiba Medical Systems, Nasu, Japan) or 3.0 T three-dimensional time-of-flight (TOF) MRA (Magnetom Verio Tim, Siemens, Erlangen, Germany; or another system: Skyra; Siemens, Erlangen, Germany) was performed for all patients.

Two neurosurgeons, blinded to the patient’s pre- and post-operative medical history, performed analysis using CTA/MRA. When a new infarction on diffusion-weighted imaging was not immediately visible after operation and clinical deterioration/neurological deficit attributable to vascular narrowing was found on MRA, CTA, or digital subtraction angiography (DSA), it was defined as SCV.

At pre-operation and the first day post-operation, CTA/MRA was performed, and again at every 3 to 7 days until day 30, based on the decision made by the neurosurgeon and neurocritical care physician. If CV was identified, daily transcranial Doppler sonography, CTA, or MRA was performed until CV was resolved. DSA was performed if CV was suspected and CTA/MRA became unsuitable for analyzing the spasm or endovascular treatment was necessary.

### Statistical analysis

SPSS 17.0 (SPSS Inc, Chicago, Illinois) was used for performing statistical analyses and *P* < 0.05 was considered significant. For continuous variables, they were shown as mean ± standard deviation, and means were analyzed by the 2-sample *t* test. For categorical variables, they were expressed as counts (percentage), and the Pearson χ^2^ test or Fisher exact test was used for their analysis. To identify predictors of SCV, multivariate logistic regression model analysis was performed. Briefly, all available variables, including those on demographic, prior medical history, admission, and aneurysm surgical variables that had univariate associations *P* < 0.25 with the outcome variable (SCV) were included in the initial pool of potential predictors. In order to remove least non-significant variables, backward stepwise regression was performed. These variables were removed one by one, until all the remaining potential predictors had *P* < 0.05. Area under the curve (AUC) model performance was calculated using the *Z* test. In this model, AUC ranges from 0.5 to 1.0, indicating predictive power from weak to strong. In order to calculate the corresponding sensitivities and specificities of the variables, the best thresholds for WFNS grades, modified Fisher grades, and Lp-PLA2 levels on admission were used. They were derived from the receiver operating characteristic (ROC) curve analyses. Using the Kaplan–Meier method, the percentage of patients surviving SCV for 30 days was calculated. After the survival curves were drawn, the log-rank test was used to compare them.

## Results

### Patient characteristics

A total of 128 patients were enrolled in this study and categorized into SCV and non-SCV groups. The age range of patients was 53.44 ± 10.51 years, with 70/128 being female. Patient demographics, prior medical history, clinical characteristics, medical complications, and admission Lp-PLA2 level were compared, and results are shown in Table 1. The average admission Lp-PLA2 level of patients with SCV was significantly higher than that of patients without SCV (Table 1).

**Table 1:** Patient characteristics by occurrence of symptomatic cerebral vasospasm

Characteristics	Total (*n* = 128)	SCV (*n* = 12)	No SCV (*n* = 116)	*P* value
Demographics				
Age, year	53.44 ± 10.51	55.00 ± 9.95	53.28 ± 10.59	0.590
Gender, female	70 (54.69%)	7 (58.33%)	63 (54.31%)	0.790
Admission clinical grade				
WFNS grade				0.007
Grade I	56 (43.75%)	1 (8.33%)	55 (47.41%)	
Grade II	6 (4.69%)	1 (8.33%)	5 (4.31%)	
Grade III	14 (10.94%)	2 (16.67%)	12 (10.34%)	
Grade IV	30 (23.44%)	3 (25.00%)	27 (23.28%)	
Grade V	22 (17.19%)	5 (41.67%)	17 (14.66%)	
Admission blood pressure				
SAP, mmHg	146.36 ± 23.97	140.08 ± 25.50	147.01 ± 23.83	0.343
DAP, mmHg	83.88 ± 11.70	81.42 ± 9.31	84.14 ± 11.92	0.445
MAP, mmHg	104.71 ± 14.34	100.97 ± 13.99	105.09 ± 14.38	0.345
Admission CT scan grade				
Modified Fisher grade	2 (2–3)	2 (2–3)	4 (3–4)	< 0.001
Medical history				
Hypertension	57 (44.53%)	7 (58.33%)	50 (43.10%)	0.312
Diabetes mellitus	16 (12.50%)	3 (25.00%)	13 (11.21%)	0.174
Cardiovascular disease	19 (14.84%)	3 (25.00%)	16 (13.79%)	0.385
Smoking history	35 (27.34%)	4 (33.33%)	31 (26.72%)	0.735
Aneurysm characteristics				
Aneurysm size, mm	7.25 ± 4.24	8.75 ± 6.22	7.09 ± 3.99	0.197
Multiple aneurysms	35 (27.34%)	3 (25.00%)	32 (27.59%)	1.000
Anterior circulation	94 (73.44%)	9 (75.00%)	85 (73.28%)	1.000
Clipping	109 (85.16%)	11 (91.67%)	98 (84.48%)	1.000
Laboratory				
Lp-PLA2, μg/L	182.09 ± 60.09	224.47 ± 76.14	177.70 ± 56.79	0.010
Medical complications				
Pneumonia	51 (39.84%)	8 (66.67%)	43 (37.07%)	0.063
Intracranial infection	11 (8.59%)	1 (8.33%)	10 (8.62%)	1.000
Sepsis	5 (3.91%)	1 (8.33%)	4 (3.45%)	0.394
Hydrocephalus	18 (14.06%)	2 (16.67%)	16 (13.79%)	0.677

Values are *n* (%), mean ± SD, median (25–75%)

*SCV* symptomatic cerebral vasospasm, *SAP* systolic arterial pressure, *DAP* diastolic arterial pressure, *MAP* mean arterial pressure, *Lp-PLA2* lipoprotein-associated phospholipase A2

### Lp-PLA2 at admission for SCV patients

For this cohort of patients, 12 (9.4%) experienced SCV within 21 days after aSAH. Both univariate and multivariate models were used to analyze variables that might be associated with SCV. These variables included WFNS grade, modified Fisher grade, diabetes mellitus, aneurysm size, and Lp-PLA2 level. The results showed that both the modified Fisher grade (OR = 10.08, 95% CI = 2.04–49.86, *P* = 0.005) and the Lp-PLA2 level (OR = 6.66, 95% CI = 1.33–33.30, *P* = 0.021) were predictors that are significantly associated with SCV (Table 2).

**Table 2:** Multivariate model analysis of SCV with admission predictors

Predictors*	Univariate analysis				Multivariate analysis^‡^	
	SCV (*n* = 12)	No SCV (*n* = 116)	OR (95% CI)	*P* value	OR (95% CI)	*P* value
WFNS grade ≥ 2^†^	11 (91.67%)	61 (52.59%)	9.92 (1.24, 79.33)	0.031		
Modified Fisher grade ≥ 3^†^	10 (83.33%)	43 (37.07%)	8.49 (1.78, 40.57)	0.007	10.08 (2.04, 49.86)	0.005
Diabetes mellitus	3 (25.00%)	13 (11.21%)	2.64 (0.63, 11.02)	0.183		
Aneurysm size ≥ 7.5 mm^†^	4 (33.33%)	46 (39.66%)	0.76 (0.22, 2.67)	0.670		
Lp-PLA2 > 169.3 μg/L^†^	10 (83.33%)	56 (48.28%)	5.36 (1.12, 25.53)	0.035	6.66 (1.33, 33.30)	0.021

*Predictors include all admission variables in Table 1 that have *P* < 0.25

^†^The cut-off point was calculated on the basis of ROC curve analysis

^‡^All variables having *P* < 0.05 from univariate analysis were included in multivariate analysis. Backward stepwise regression methods were performed to create the final model, whereby the least nonsignificant variable was removed from the model one at a time, until all remaining variables had *P* < 0.05

**Fig. 1:**
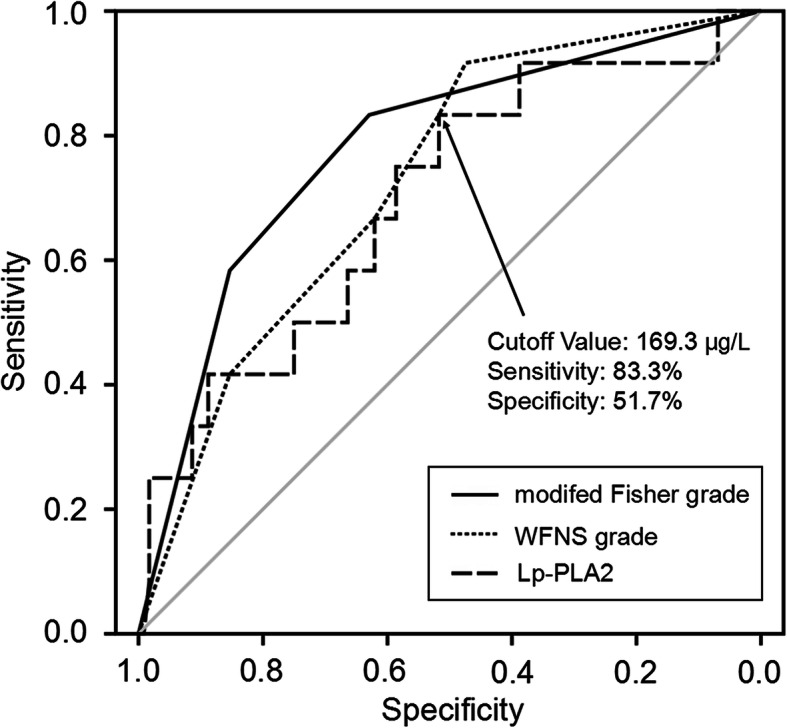
Comparisons of AUC for identifying SCV using Lp-PLA2, the WFNS grade, and modified Fisher grade. ROC curves were constructed on the basis of the sensitivity and specificity of the WFNS grade, modified Fisher grade, and Lp-PLA2 for identifying SCV. *Z* test was used for comparing AUC performances and results revealed that the predictive performance of the Lp-PLA2 was similar to that of the WFNS grade (*Z* = 0.280, *P* = 0.780) and modified Fisher grade (*Z* = 0.751, *P* = 0.452).

The Lp-PLA2 level for assessing the predictive performance of SCV is represented as AUC = 0.696 (95% CI = 0.536–0.856), as revealed by ROC curve analysis. Using the best threshold of 169.3 μg/L for Lp-PLA2, the sensitivity was derived as 83.3% and the specificity was derived as 51.7% (Fig. 1). The predictive performance of the Lp-PLA2 was similar to that of WFNS grade (AUC = 0.726, 95% CI = 0.591–0.862; *Z* = 0.280, *P* = 0.780) and modified Fisher grade (AUC = 0.778, 95% CI = 0.637–0.920; *Z* = 0.751, *P* = 0.452) in aSAH (Fig. 1).

### The non-SCV rate analysis of patients with different WFNS grades with and without elevated Lp-PLA2

**Fig. 2:**
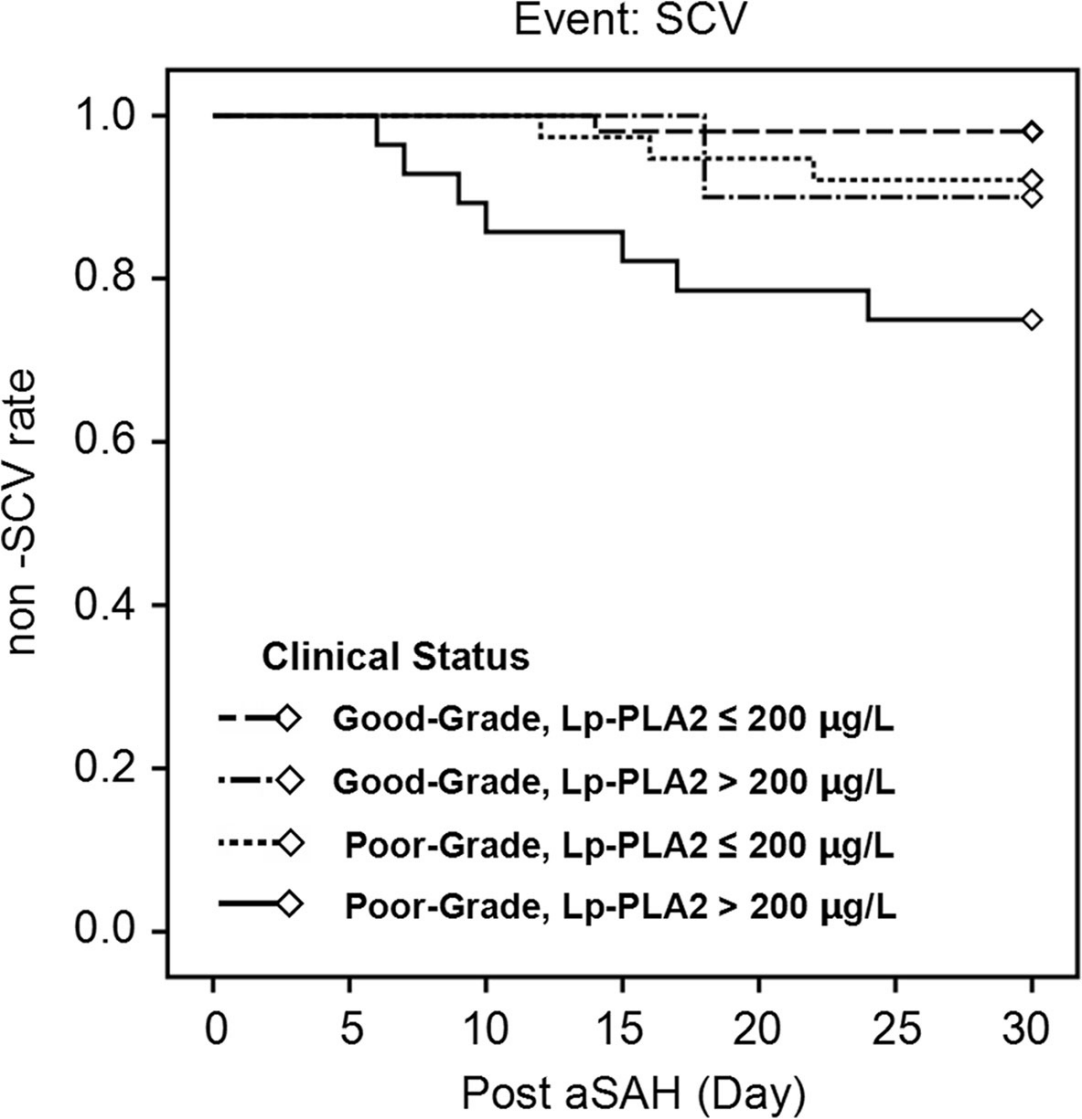
The Kaplan–Meier curve of the 30 days non-SCV rate of patients with and without elevated admission Lp-PLA2. Post hoc log-rank testing revealed that the good WFNS grade patients having poor Lp-PLA2 level (> 200 μg/L) had a similar non-SCV rate to that of good WFNS grade patients having good Lp-PLA2 level (≤ 200 μg/L) (*P* = 0.712), but the poor WFNS grade patients having poor Lp-PLA2 level had significantly lower non-SCV rate than poor WFNS grade patients having good Lp-PLA2 level (*P* = 0.001)

ROC curve analysis showed that the predictive performances of the Lp-PLA2 and WFNS grade were similar. We further analyzed the relationship between the Lp-PLA2 and the occurrence of SCV in patients with different WFNS grades. The Kaplan–Meier curve shows a 30 days non-SCV rate of 75% (21/28) for poor WFNS grade patients with poor Lp-PLA2 level (> 200 μg/L), of 92.1% (35/38) for poor WFNS grade patients with good Lp-PLA2 level (≤ 200 μg/L), of 90.0% (9/10) for good WFNS grade patients (admission WFNS grade < 3) with poor Lp-PLA2 level, and of 98.1% (51/52) for good WFNS grade patients with good Lp-PLA2 level. *P* = 0.006 as calculated in the log-rank test. Post hoc log-rank testing revealed that in the good WFNS grade patient sub-group, patients with poor Lp-PLA2 level had a similar non-SCV rate to that of patients with good Lp-PLA2 level (*P* = 0.196); but in the poor WFNS grade patient sub-group, patients with poor Lp-PLA2 level had significantly lower non-SCV rate than that of patients having good Lp-PLA2 level (*P* = 0.045) (Fig. 2).

## Discussion

The current study demonstrates that the average Lp-PLA2 level of patients with SCV was significantly higher than that of patients without SCV. Furthermore, in multivariate logistic regression models of finding predictors of SCV, Lp-PLA2 level was an independent and significant predictor after adjustment for confounders, including the WFNS grade, diabetes mellitus, and aneurysm size. Currently, among all grading schemes, the modified Fisher grade and the WFNS grade are the most widely used, because of their relatively strong predictive power and ease of use [4–6]. It was found that for SCV patients, the predictive performance of Lp-PLA2 was comparable with the WFNS grade and modified Fisher grade, which indicates the potential of the Lp-PLA2 level as a new marker in aSAH prognosis.

As one of the worst and most common complications of aSAH, CV usually occurs within 1 month post-aSAH and is associated with unfavorable outcome [26]. Despite improvements in early CT diagnosis and neurosurgical critical care, approximately 30–70% of aSAH patients will experience ACV, approximately 10–30% of aSAH patients will experience SCV, and nearly 15% patients will die or have devastating neurologic outcomes [20, 27, 28]. Currently, the pathogenesis and etiology of CV are still poorly understood. Although there are a wide variety of treatments to prevent post-aSAH CV, their effectiveness differs significantly. The treatment of CV via medication is usually suboptimal and often associated with cerebral ischemia or infraction, further worsening the condition of patient. Therefore, if early prognosis of CV could be achieved to predict potential risks, optimized care and more effective distribution of healthcare resources might be made possible for these patients. There have been studies reporting on the connection between elevated Lp-PLA2 level and ACV in aSAH patients [19]. The associated morbidity and mortality of SCV could be higher than ACV. However, whether Lp-PLA2 can predict the occurrence of SCV in aSAH patients is still unclear.

Based on published literature, the possible causes of post-aSAH CV include endothelial cell interactions, alterations in calcium signaling, oxidative processes, spreading depolarization, and/or inflammation [29, 30]. For goal-oriented therapy, the aim is to create an optimal physiological environment for the comatose injured brain, and technologies such as detection of related biomarkers may eventually be used. Biomarker measurements, usually quantitative, can be utilized in diagnosis and monitoring of patient response to treatment. Although there exist established biomarkers of predicting aSAH, there is a need for better use of them and more reliable ones, so a more effectively guided clinical diagnosis and assessment of aSAH can be achieved [12–14]. Biomarkers such as C-reactive protein, which are related to inflammatory reaction, can be used to predict CV after aSAH [31, 32]. Compared to C-reactive protein, which has high-sensitivity, LP-PLA2 could be more specific when predicting inflammation in vessels [33]. More and more animal and human studies have been done to explore clinical application of Lp-PLA2. Results showed that the plasma LP-PLA2 level is positively related to oxidative stress, atherosclerosis, and various cerebrovascular and cardiovascular events including ischemic and hemorrhagic strokes [17, 18, 34].

The rate of SCV was 9.4% in our cohort of patients. The levels of the LP-PLA2 within the first 24 h were significantly higher in our cohort of aSAH patients who experienced SCV compared to those who did not. In the multivariate logistic regression model analysis, LP-PLA2 was an independent and significant predictor of SCV. Based on past research [25], poor Lp-PLA2 level indicates moderate inflammation of the vessels and an increased risk of cardiovascular disease. In this study, the best thresholds of Lp-PLA2 level used in predicting SCV were lower than 200 μg/L. Using lower Lp-PLA2 level to predict SCV improved sensitivity but decreased specificity. However, considering the severity of SCV, predicting SCV using biomarkers with high sensitivity might be helpful for identifying more patients at high risk of SCV. Using the best threshold of 169.3 μg/L, the specificity of Lp-PLA2 level for predicting SCV was calculated to be 51.7%, and the sensitivity was 83.3%, which is a relatively high for diagnostic purpose (> 80%).

The clinical grading schemes for assessing severity of aSAH and interpretation of CT scans, such as the WFNS grade and modified Fisher grade [4–6], are often used to predict occurrence of CV. However, identification of more easily measurable biomarkers for predicting CV would still be helpful for prognosis and risk mitigation. Lp-PLA2 is secreted by endothelium inflammatory cells and then released into the blood, and it is a specific marker that reflects degree of inflammation of the vessel endothelium. It can be easily obtained and integrated into daily practice. Compared to subjective clinical grades, Lp-PLA2 might be more objective and might be able to distinguish changes in the physical condition of a patient that may be missed when using clinical grades, which could lead to modifications in the treatment plan. In our study, the ROC curve analysis revealed that the predictive performance of the Lp-PLA2 was comparable to that of WFNS grade. Therefore, we further analyzed the relationship between Lp-PLA2 and the occurrence of SCV in patients with different WFNS grades. It was found that the elevated admission LP-PLA2 level may have clinical value especially for patients with poor WFNS grade (WFNS grade ≥ 3). For good WFNS grade patients, those with poor Lp-PLA2 level (> 200 μg/L) and with good Lp-PLA2 level (≤ 200 μg/L) had similar non-SCV rate; but for poor WFNS grade patients, those with poor Lp-PLA2 level had significantly lower non-SCV rate than patients with good Lp-PLA2 level (*P* = 0.045). This result suggests that Lp-PLA2 might be used in combination with WFNS in predicting the SCV. These patients with poor WFNS grade and admission Lp-PLA2 level > 200 μg/L might need more careful post-surgery monitoring.

There are still a number of limitations of this study, including its observational design. First, ELISA, the most common method of detecting level of Lp-PLA2 was used in our study. However, this level might change when a different measurement method is used [35]. Therefore, one of the key issues needs to be addressed is the standardization of Lp-PLA2 measurement method. Second, not many SCV cases were included in this study, and more data with higher number of enrolled patients are still needed to confirm findings in this study.

## Conclusions

In this study, it was found that the Lp-PLA2 level obtained within the first 24 h post-aSAH was associated with SCV following aSAH. The Lp-PLA2 might be helpful as a marker easily obtained in the clinical setting for predicting SCV in patients with aSAH. In order to determine if changes in Lp-PLA2 over time and the onset of SCV are related to each other, further research is still needed.

## Data Availability

All data generated or analyzed during this study are included in the article.

## Abbreviations

aSAH: Aneurismal subarachnoid hemorrhage
Lp-PLA2: Lipoprotein-associated phospholipase A2
SCV: Symptomatic cerebral vasospasm
WFNS: World Federation of Neurosurgical Societies
ACV: Angiographic cerebral vasospasm
CTA: Computed tomography angiography
DSA: Digital subtraction angiography
GCS: Glasgow coma scale
ELISA: Enzyme-linked immunosorbent assay
COV: Coefficient of variation
TOF: Time-of-flight
AUC: Under the curve
ROC: Receiver operating characteristic
